# *DDX39B* (*BAT1*), *TNF* and *IL6* gene polymorphisms and association with clinical outcomes of patients with *Plasmodium vivax* malaria

**DOI:** 10.1186/1475-2875-13-278

**Published:** 2014-07-19

**Authors:** Vitor RR Mendonça, Ligia CL Souza, Gabriela C Garcia, Belisa ML Magalhães, Marcus VG Lacerda, Bruno B Andrade, Marilda S Gonçalves, Manoel Barral-Netto

**Affiliations:** 1 Centro de Pesquisas Gonçalo Moniz, Fundação Oswaldo Cruz (FIOCRUZ), Salvador, Brazil; 2 Faculdade de Medicina, Universidade Federal da Bahia, Salvador, Brazil; 3 Fundação de Medicina Tropical Dr Heitor Vieira Dourado, Manaus, Brazil; 4 Immunobiology Section, Laboratory of Parasitic Diseases, National Institute of Allergy and Infectious Diseases, National Institutes of Health, Bethesda, MD, USA; 5 Instituto de Investigação em Imunologia, Instituto Nacional de Ciência e Tecnologia, São Paulo, Brazil

**Keywords:** *DDX39B* (*BAT1*), Single nucleotide polymorphisms, Immune response, *Plasmodium vivax*, Malaria

## Abstract

**Background:**

*DDX39B (BAT1)* encodes an RNA helicase known to regulate expression of TNF and IL-6. Elevated levels of these two cytokines are associated with increased severity of clinical malaria. The aim of this study was to investigate the relationship between single nucleotide polymorphisms (SNPs) in the *DDX39B*, *TNF* and *IL6* genes and the clinical outcomes of patients with *Plasmodium vivax* malaria.

**Methods:**

Cross-sectional investigations were carried out in two regions of the Brazilian Amazon where several studies on the pathogenesis of vivax malaria had been performed. Individuals were categorized according to infection status as well as clinical presentation into the following groups: uninfected, asymptomatic infection, mild infection, or complicated infection. Polymorphisms were identified using PCR restriction fragment-length polymorphism analysis and the restriction enzymes *Nl*aIII or *Nco*I. The plasma levels of cytokines were determined using ELISA.

**Results:**

The G allele of *DDX39B-*22C > G was associated with absent or decreased manifestations of malaria and the C allele was a risk factor for disease complications. Study participants heterozygous for *TNF-308* (GA) and *DDX39B-348* (CT) had higher TNF levels than wild-type participants. Haplotypes that included *DDX39B* (-22C > G and -348C > T) and *TNF* polymorphisms were not directly associated with mild or complicated malaria infections; however, haplotypes AGC, ACC, GGT, AGT and ACT were associated with increased TNF levels. Participants with genotype combinations GC/CC/GG/GG and GG/CT/GG/GG (*DDX39B-22*/*DDX39B-348*/*TNF-308*/*IL6-176*) had decreased and increased risk of mild malaria, respectively, compared with asymptomatic and uninfected participants. GC/CC/GG/GG was linked to decreased TNF and IL-6 levels.

**Conclusions:**

This is the first study to describe patients with *DDX39B* and *IL6* SNPs who had vivax malaria. These findings support the postulation that a set of mutations in immune-related genes is associated with inflammatory mediators and the clinical outcomes of patients with malaria.

## Background

*Plasmodium vivax* malaria is a major worldwide threat, with two to three billion people remaining at risk of infection [1]. The clinical outcomes of patients with vivax malaria range from asymptomatic infection to complicated, and potentially lethal disease [2]. The risk for progression of severe *Plasmodium falciparum* malaria has been thought to be partly accounted for by host genetic factors [3]. These genetic factors are likely to involve several genetic alterations in immune mediators as well as molecules involved in mechanisms of cytoadherence and haemoglobinopathies [4]. Understanding the key host genetic determinants of susceptibility to malaria is critical for developing better therapies and vaccine strategies.

The nuclear protein HLA-B-associated transcript 1 (BAT1) is an RNA helicase encoded by the *DDX39B* gene (DEAD [Asp-Glu-Ala-Asp] box polypeptide 39B, also known as *BAT1*). BAT1 has been described as a negative regulator of inflammation by modulating expression of proinflammatory cytokines, such as TNF and IL-6, which suggests that it plays a protective role in several immune-mediated disorders [5]. The -22C > G and -348C > T polymorphisms in the promoter region of *DDX39B* have been shown to affect its transcriptional activity and the binding of nuclear transcription factors such as YY1 and Oct1 oligonucleotides at the respective positions -348 and -22 relative to the transcription start site of *DDX39B*[6]. TNF (=TNFalpha or TNFA) and IL-6 are key mediators associated with malaria symptoms, and their levels are increased in proportion to the severity of disease [7-12]. The hypothesis that *DDX39B* may have an effect on the clinical presentation of malaria by its modulation of the expression of proinflammatory cytokines involved in the pathogenesis of the disease is appealing, but has not yet been tested.

Single nucleotide polymorphisms (SNPs) in the *TNF* (-308G > A) and *IL6* (-176G > C) genes may regulate the plasma levels of these cytokines; however, the mechanism of regulation is not yet fully understood [13-15]. Given the major role of the host immune system in *P. vivax* infection, the aim of this study was to determine whether mutations in the *DDX39B*, *TNF* and *IL6* genes were associated with the clinical outcomes of patients with vivax malaria. The frequency of SNPs in *DDX39B* (-22C > G and -348C > T), *TNF* (-308G > A) and *IL6* (-176G > C) were compared between *P. vivax*-infected study participants who exhibited different clinical outcomes, including asymptomatic infection, mild malaria, complicated malaria, and no infection. Associations between these immune-related mutations and plasma levels of TNF, IL-6, C-X-C motif chemokine 10 (CXCL10), and C-reactive protein (CRP) were also tested. The results reported here revealed that a combination of *DDX39B, TNF* and *IL6* host genotypes were associated with manifestations of malaria, mainly by altering plasma levels of TNF and IL-6.

## Methods

### Study participants

This report describes series of patients from two distinct studies. The first study performed retrospective analyses of cryopreserved heparinized blood samples from participants living in riverine communities of the state of Rondônia, in the Brazilian Western Amazon, who were recruited between 2006 and 2007, as previously described [7,16-23]. Malaria was diagnosed using two methods: 1) microscopic examination of a thick blood smear performed by professionals at the Brazilian National Foundation of Health (FUNASA); and 2) polymerase chain reaction (PCR) performed at the Oswaldo Cruz Foundation (FIOCRUZ), Salvador, Brazil, as previously described [16-18]. The study included individuals who had been living in the endemic area for more than six months. Exclusion criteria included conditions known to interfere with the parameters evaluated in this report, such as coinfections and chronic diseases: *P. falciparum* infection confirmed by nested PCR; documented or referred viral hepatitis (hepatitis A, B, C, D virus [HAV, HBV, HCV, HDV]); chronic alcoholism; human immunodeficiency virus (HIV) infection; yellow fever; dengue; leptospirosis; tuberculosis; Hansen disease; visceral leishmaniasis; cancer and/or other chronic degenerative disease; sickle cell trait; and the use of hepatotoxic and immunosuppressant drugs. A total of 257 participants were enrolled in this first part of the study. As reported previously, all asymptomatic participants infected with *P. vivax* who were identified by active case detection were monitored for 30 days for the evaluation of malaria manifestations [16-18]. Participants who were positive for *P. vivax* infection but remained without acute febrile signs for 30 days were considered to be asymptomatic cases. Those individuals with positive parasitaemia and with mild symptoms were considered to have mild vivax malaria. Thus, the individuals from this region were divided into three groups as follows: mild malaria (n = 76), asymptomatic malaria (n = 104) and uninfected controls (n = 77). All symptomatic cases were treated following the guidelines of the National Foundation of Health, Brazil, and received chloroquine for three days and primaquine (0.5 mg/kg/day) for seven days. The asymptomatic carriers were treated after the monitoring period, as reported previously [17]. This first part of the study was approved by the Ethics Committee of the São Lucas University, Rondônia, Brazil; and all participants provided written informed consent.

### Participants with complicated *Plasmodium vivax* malaria

Because there was a small number and unavailable blood samples of malaria cases (n = 9) with signs/symptoms of complicated disease among the first series of patients recruited in Rondonia, in order to evaluate the role of *DDX39B (BAT1)* polymorphism in complicated cases of vivax malaria, a second series of patients (second part of this report) was recruited from the state of Amazonas in the Western Brazilian Amazon between 2009 and 2013. Individuals of all ages who were hospitalized with an unidentified acute febrile syndrome at the reference hospital from the Fundação de Medicina Tropical Dr Heitor Vieira Dourado (FMT-HVD), Amazonas, Brazil, were tested for malaria using microscopic examination of a thick blood thick smear, and those with PCR-confirmed *P. vivax* were recruited. Patients were excluded for the following conditions: microscopic or molecular diagnosis of *P. falciparum* or *P. vivax* and *P. falciparum* malaria (mixed infection), serologic diagnosis of viral hepatitis (HAV, HBV, HCV, and HDV), HIV, or leptospirosis. Patients with vivax malaria with primaquine-induced haemolysis (patients taking primaquine with decreasing haemoglobin levels to <10 g/dL and reticulocyte counts >1.5%, or increased indirect bilirubin levels after primaquine treatment) were also excluded. The study participants from this second series had either mild signs/symptoms of acute malaria (mild malaria, n = 69) or clinical complications, as listed in Table 1 (complicated vivax malaria, n = 31). Complicated vivax malaria was defined according to the criteria for severe malaria from the World Health Organization (WHO) or based on the presence of hyperbilirubinaemia (serum total bilirubin > 51.3 μmol/L) [24]. Although all the participants in this second study were PCR positive, some of these study patients with complicated malaria (just for this particular group) had already started therapy for malaria before blood samples were taken, which might have affected the results of the laboratory and parasitaemia evaluations shown in Table 1. This second part of the study was approved by the Ethics Committee of the FMT-HVD, and all participants provided written informed consent.

**Table 1:** Characteristics of the second series of participants with complicated vivax malaria: Amazonas, Brazil

			**Clinical presentation at admission**						
**Patient no.**	**Gender**	**Age (years)**	**Major manifestation**	**Secondary manifestation**	**Previous treatment**	**Parasitaemia**	**Disease duration (days)**	**Haemoblogin (g/dL)**	**Total bilirubin (μmol/L)**
1	M	59	hyperbilirubinaemia		No	6,222	7	16.9	403.56
2	F	38	hyperlactemia		Yes	0	10	10.9	30.78
3	M	31	hyperbilirubinaemia		No	3,298	10	11.0	54.72
4	F	37	hyperbilirubinaemia		No	67,467	7	8.9	51.30
5	M	18	hyperbilirubinaemia		No	1,848	4	13.5	165.87
6	M	45	severe anaemia	hyperbilirubinaemia	No	243	10	6.5	138.51
7	F	40	severe anaemia		No	33,553	10	7.0	27.36
8	M	66	convulsion		Yes	185	14	13.0	*
9	M	17	hyperbilirubinaemia		No	226	6	11.9	53.01
10	M	31	hyperbilirubinaemia		No	5,937	15	13.5	165.87
11	M	1	severe anaemia		No	38,712	9	5.0	8.55
12	M	26	hyperbilirubinaemia		No	128	2	10.6	61.56
13	F	4 months	convulsion	prostration	No	108,033	7	11.7	*
14	M	19	severe anaemia		No	0	17	6.5	15.39
15	M	50	respiratory failure		No	310	5	10.1	8.55
16	F	35	severe anaemia	hyperbilirubinaemia	No	34,673	1	4.8	138.51
17	F	41	respiratory failure		No	24,815	8	9.9	39.33
18	F	18	hyperbilirubinaemia		No	670	5	11.7	61.56
19	M	44	severe anaemia		Yes	0	7	6.9	13.68
20	F	23	severe anaemia		Yes	264	8	6.4	6.84
21	M	5	prostration		No	291	15	8.2	6.84
22	M	21	hyperbilirubinaemia		Yes	832	7	9.7	102.6
23	M	12	respiratory failure		Yes	0	6	7.8	15.39
24	F	74	respiratory failure		No	325	5	11.7	29.07
25	M	37	respiratory failure		Yes	0	10	7.1	17.10
26	F	26	hyperbilirubinaemia		No	254	9	11.3	140.22
27	M	53	hyperbilirubinaemia		No	45,833	6	11.8	59.85
28	F	36	hyperbilirubinaemia		No	0	3	7.9	85.50
29	F	15	severe anaemia		Yes	8,894	8	6.9	11.97
30	M	22	hyperbilirubinaemia		No	3,054	7	15.1	73.53
31	M	25	hyperbilirubinaemia		Yes	27,954	3	15.0	138.51

Severe anaemia was defined as haemoglobin levels below 7 g/dL for adults and below 5 g/dL for children and hyperbilirubinaemia by serum total bilirubins >51.3 μmol/L. Respiratory failure was defined as tachypnea, shortness of breath, mental confusion clinical signs of hypoxaemia (central and/or peripheral cyanosis). Previous treatment indicates participants who already started malaria therapy before the blood sample collection. *Total bilirubin was not measured in these individuals.

### Genotyping

DNA was extracted from 200 μL of peripheral blood using a standard Qiagen DNA blood mini kit (Valencia, CA, USA) according to the manufacturer’s protocol. The SNPs at positions -22 (C > G) (rs2239527; G ancestral allele) and -348 (C > T) (rs2239528 ;C ancestral allele) in the promoter region of *DDX39B* and at position -176 of *IL6* (G > C) (rs2234683; G ancestral allele) were typed using PCR restriction fragment-length polymorphism analysis with the restriction enzyme *Nla*III (New England Biolabs), according to protocols previously published by Ramasawmy *et al.*[25] and Yalcin *et al.*[26], respectively. *TNF-308* (G > A) (rs1800629; G ancestral allele) polymorphism was also evaluated using PCR restriction fragment-length polymorphism analysis with the restriction enzyme *Nco*I (New England Biolabs), as published previously [27]. *DDX39B* and *TNF* PCR products were electrophoresed on 10% polyacrylamide gels and *IL6* products were separated by electrophoresis on 1% agarose gels under nondenaturing conditions. The PCR products were then detected by staining with ethidium bromide and visualized under ultraviolet illumination.

### Plasma measurements

The plasma levels of IL-6, CXCL10, and TNF were measured using a cytometric bead array system (BD Biosciences Pharmingen, Franklin Lakes, NJ, USA) according to the manufacturer’s protocol. CRP levels in plasma were measured using the turbidimetric immunoassay method, performed at the Federal University of Bahia and Faculdade São Lucas, Brazil.

### Statistical analysis

Categorized variables (genotypes, alleles, haplotypes, and genotype combinations) were compared using the Chi-square test or Fisher exact test in 2x2 contingency tables along with the relevant odds ratio (OR) and 95% confidence interval (CI). Univariate linear regression analysis was performed to assess the associations between combinations of genotypes and malaria symptomatology. Ordinal variables were evaluated using the Mann–Whitney (between two groups) or Kruskal-Wallis test followed by the Dunn multiple comparison test or trend analysis (when more than two groups were compared). Hardy-Weinberg equilibrium (HWE) was assessed for the different groups by comparing the observed number of different genotypes with those expected under HWE for the estimated allele frequency. The power of this study was calculated based on a medium effect size, a significance level of 0.05, and four degrees of freedom (Chi-square test). A power of 98.3% was found for the first series of participants (n = 257) and a power of 71.10% for the second series of participants (n = 110). Statistical analyses were performed using GraphPad Prism (version 5.0b) software (GraphPad Software, San Diego, CA, USA) or R version 2.15.1 (The R Foundation for Statistical Computing, Vienna, Austria).

## Results

### Baseline characteristics of first series of participants

All different groups of vivax malaria infection had a slightly majority of women. Participants with asymptomatic malaria were older than the uninfected participants and those with mild malaria (*P* = 0.0264; Table 2). Participants with asymptomatic malaria reported a higher number of previous infections (median 15.0, interquartile range (IQR) 12.00-18.75) than the uninfected participants or those with mild infection (*P* < 0.0001; Table 2). In addition, participants with asymptomatic *P. vivax* infection reported living for a longer time in the endemic area (71.15% more than ten years) than those in the other groups (*P* = 0.0030; Table 2). These results were expected, since this set of patients was a subsample of a larger cohort of individuals where similar results were found [7,16-23].

**Table 2:** Baseline characteristics of the first series of participants enrolled in the first part of the study: Rondonia, Brazil

	**Uninfected (n = 77)**	**Asymptomatic vivax malaria (n = 104)**	**Mild vivax malaria (n = 76)**	***P *** **value**
**Male - no. (%)**	36 (46.75)	47 (45.19)	36 (47.37)	0.9548**
Median (IQR*) age (yr)	35.00 (25.50-45.00)	42.00 (32.00-49.00)	36.00 (27.25-50.00)	0.0264***
Median (IQR*) of previous malaria episodes	12.00 (6.00-17.00)	15.00 (12.00-18.75)	6.00 (1.00-12.75)	<0.0001***
**Time residing in the area (yr)**				
≤2	17 (22.08)	24 (23.08)	24 (31.58)	0.0011**
3 to 10	19 (24.67)	6 (5.77)	16 (21.05)	
>10	41 (53.25)	74 (71.15)	36 (47.37)	

* IQR, interquantile range; **Categorized variables were compared using Chi-square test; ***Ordinal variables were compared using the Kruskal-Wallis test with the Dunn multiple comparison test

### Single nucleotide polymorphisms

Genotype and allele distributions of *IL6*-176G > C and *TNF*-308G > A polymorphisms were compared in the main clinical groups and in participants stratified according to mild *vs* asymptomatic vivax malaria infection, infected *vs* uninfected and asymptomatic *vs* uninfected (Table 3). No association was found between alleles or genotypes of *IL6* and *TNF* SNPs and the different clinical outcomes of vivax malaria (Table 3). The distributions of *IL6*-176G > C and *TNF*-308G > A genotypes in all categories were under HWE. Table 4 shows the results for *DDX39B* polymorphisms (-22C > G and -348C > T) according to the same categories of participants (Table 4). No differences were observed for the SNPs *DDX39B-22C > G* and *DDX39B-348C > T* with regard to mild malaria, asymptomatic malaria, or infection status. The distribution of *DDX39B-*22C > G in the asymptomatic participants was not under HWE (x2 = 6.10, *P* = 0.0134), suggesting those participants reflect evolutionary selective pressure. The frequencies of *DDX39B* genotypes were under HWE in all the other categories of vivax malaria infection.

**Table 3:** ***TNF *** **(-308G > A) and *IL-6 *** **(-176G > C) polymorphisms and outcome of vivax malaria infection: first series of participants from Rondonia, Brazil**

	**Genotype**			**χ2**	**Allele frequency**		**χ2**	**GG *vs *** **GA + AA χ2**
	**GG n (%)**	**GA n (%)**	**AA n (%)**		**G (%)**	**A (%)**		
***TNF-308 *** **polymorphism**								
Uninfected (n = 77)	60 (77.92)	15 (19.48)	2 (2.60)	2.12 p = 0.7134^#^	135 (87.66)	19 (12.34)	0.57 p = 0.7509	0.96 p = 0.6178
Asymptomatic malaria (n = 104)	78 (75.00)	25 (24.04)	1 (0.96)		181 (87.02)	27 (12.98)		
Mild malaria (n = 76)	54 (71.05)	21 (27.63)	1 (1.32)		129 (84.87)	23 (15.13)		
Infected *vs* uninfected				p = 0.3401*^#^			p = 0.6745*	P = 0.5316*
Mild *vs* asymptomatic				p = 0.6059*^#^			p = 4798*	P = 0.4323*
Asymptomatic *vs* uninfected				p = 0.5866*^#^			p = 8749*	P = 0.7252*
	**Genotype**			**χ2**	**Allele frequency**		**χ2**	**GG *vs *** **GC + CC χ2**
	**GG n (%)**	**GC n (%)**	**CC n (%)**		**G (%)**	**C (%)**		
***IL6-176 *** **polymorphism**								
Uninfected (n = 77)	49 (63.64)	23 (29.87)	5 (6.49)	2.16 p = 0.7058	121 (78.57)	33 (21.43)	1.61 p = 0.4462	1.91 p = 0.3854
Asymptomatic malaria (n = 104)	60 (57.69)	38 (36.54)	6 (5.77)		158 (75.96)	50 (24.04)		
Mild malaria (n = 76)	40 (52.63)	30 (39.47)	6 (7.90)		110 (72.37)	42 (27.63)		
Infected *vs* uninfected				1.56 p = 0.4579			p = 0.3695*	p = 0.2702*
Mild *vs* asymptomatic				1.30 p = 0.5214			p = 0.2618*	p = 0.2711*
Asymptomatic *vs* uninfected				0.88 p = 0.6435			p = 0.0819*	p = 0.4458*

*In these cases, Fisher exact test was used. ^#^Analysis excluded genotype AA. χ2: coefficient and *P* value measured using Chi-square test. Infected individuals represent symptomatic plus asymptomatic cases.

**Table 4:** ***DDX39B *** **polymorphisms (-22C > G and -348C > T) and outcome of vivax malaria infections: first series of participants from Rondonia, Brazil**

	**Genotype**			**χ2**	**Allele frequency**		**χ2**	**CC *vs *** **CG + GG χ2**
	**CC n (%)**	**CG n (%)**	**GG n (%)**		**C (%)**	**G (%)**		
***DDX39B-22 *** **polymorphism**								
Uninfected (n = 77)	10 (12.99)	38 (49.35)	29 (37.66)	6.75 p = 0.1496	58 (37.66)	96 (62.34)	2.35 p = 0.3088	0.53 p = 0.7660
Asymptomatic malaria (n = 104)	15 (14.42)	64 (61.54)	25 (24.04)		94 (45.19)	114 (54.81)		
Mild malaria (n =7 6)	13 (17.10)	34 (44.74)	29 (38.16)		60 (39.47)	92 (60.53)		
Infected *vs* uninfected				1.48 p = 0.4758			p = 0.3281*	p = 0.2460*
Mild *vs* asymptomatic				2.90 p = 0.2339			p = 0.6244*	p = 0.7028*
Asymptomatic *vs* uninfected				3.98 p = 0.1364			p = 0.1625*	p = 0.8307*
	**Genotype**				**Allele frequency**			**CC *vs *** **TC + TT χ2**
	**CC n (%)**	**TC n (%)**	**TT n (%)**		**C (%)**	**T (%)**		
***DDX39B-348 *** **polymorphism**								
Uninfected (n = 77)	64 (83.12)	12 (15.58)	1 (1.30)	1.99 p = 0.3691^#^	140 (90.91)	14 (9.09)	1.00 p = 0.6041	1.56 p = 0.4586
Asymptomatic malaria (n = 104)	83 (79.81)	21 (20.19)	0 (0.00)		187 (89.90)	21 (10.10)		
Mild malaria (n = 76)	57 (75.00)	19 (25.00)	0 (0.00)		133 (87.50)	19 (12.50)		
Infected *vs* uninfected				p = 0.2397*^#^			p = 0.5342*	p = 0.4012*
Symptomatic *vs* asymptomatic				p = 0.2372*^#^			p = 0.3469*	p = 0.3108*
Asymptomatic *vs* uninfected				p = 0.5594*^#^			p = 0.8578*	p = 0.7008*

*In these cases, Fisher exact test was used. ^#^Analysis excluded genotype TT. χ2: coefficient and *P* value measured using Chi-square test. Infected individuals represent symptomatic plus asymptomatic cases.

### Association of single nucleotide polymorphisms with inflammatory mediators levels

**Figure 1:**
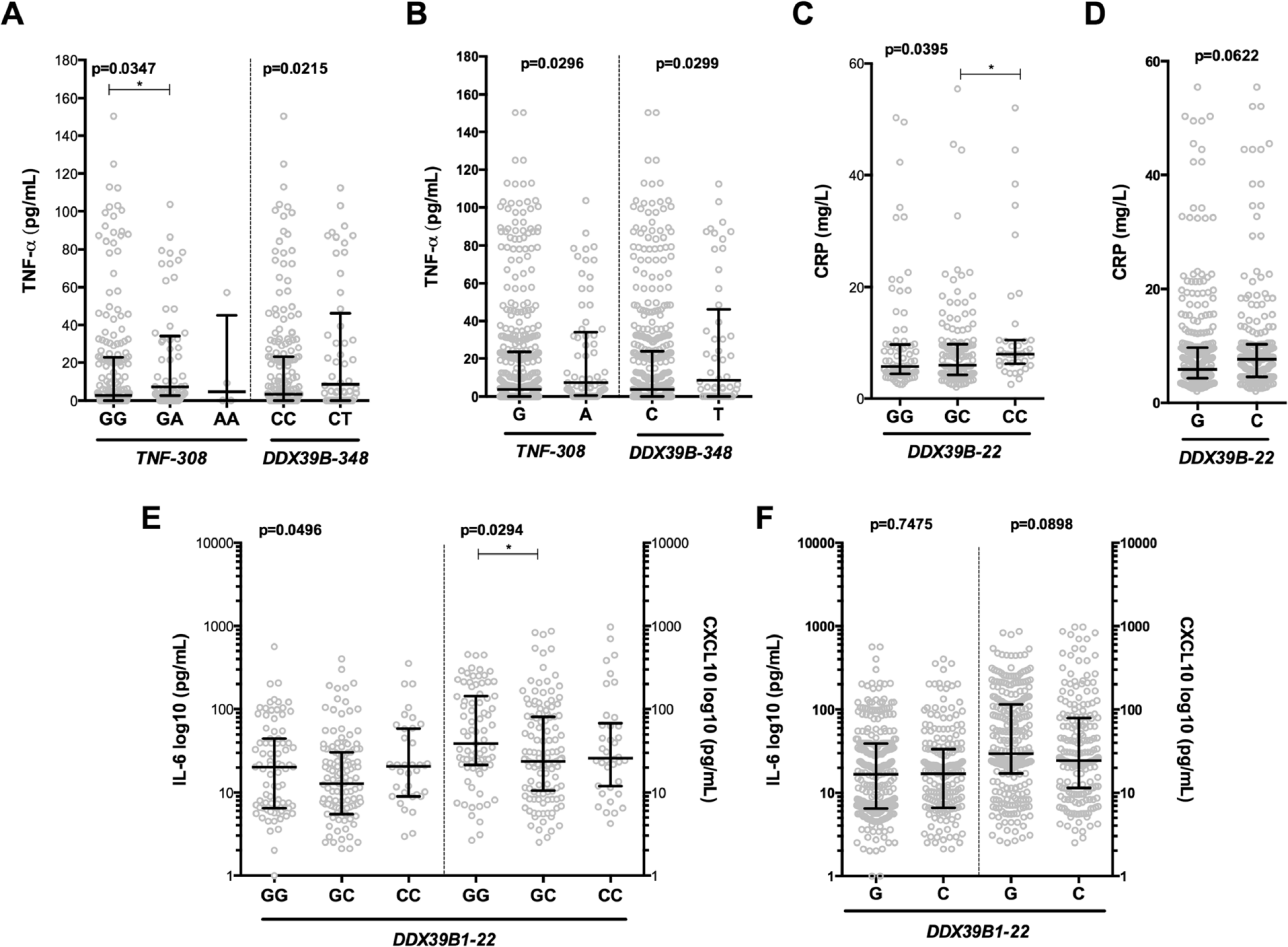
**Influence of genetic alterations on levels of inflammatory mediators.** Each symbol represents a single patient, and the lines represent medians and interquartile range. **(A)** Levels of TNF according to genotypes of *TNF-*308G > A and *DDX39B*-348C > T polymorphisms. Analysis excluded TT (*DDX39B*-348C > T), because only 1 uninfected participant had this genotype. **(B)** Levels of TNF according to alleles of *TNF*-308G > A and *DDX39B*-348C > T polymorphisms. **(C)** CRP plasma levels according to different *DDX39B*-22C > G genotypes. **(D)** CRP plasma levels according to different *DDX39B*-22C > G alleles. **(E)** IL-6 and CXCL10 levels according to *DDX39B*-22C > G genotypes. **(F)** IL-6 and CXCL10 levels according to *DDX39B*-22C > G alleles. The data were compared using the Mann–Whitney test (comparisons between two groups), the Kruskal-Wallis test with Dunn multiple comparisons or linear trend analysis (comparisons between more than 2 groups). *P* values are shown in each panel.

The SNPs were assessed regarding association with systemic levels of TNF, IL-6, CXCL10, and CRP, which have been associated with vivax malaria manifestations [23]. Participants with the *TNF-*308 GA genotype or A allele had higher levels of TNF than those with the GG genotype or G allele (*P* = 0.0347 and *P* = 0.0296, respectively, Figure 1A,B). Participants with the *DDX39B*-348 CT genotype or T allele had higher concentrations of TNF than those with the CC genotype or C allele (*P* = 0.0215 and *P* = 0.0299, respectively, Figure 1A,B). Polymorphism at *DDX39B*-22 appeared to correlate with serum concentrations of CRP and CXCL10, but not IL-6. Participants with the *DDX39B*-22 CC genotype exhibited higher levels of CRP than those with the GC genotype (*P* = 0.0395, Figure 1C) but this difference was not observed in the allele analysis (*P* = 0.0622, Figure 1D). No relevant associations were found between genotypes or alleles of *DDX39B-*22 C > G and plasma IL-6 levels (*P* = 0.0496 and *P* = 0.7475, respectively, Figure 1E,F). Furthermore, participants carrying the *DDX39B-*22 CG genotype had lower CXCL10 levels than those with the GG genotype (*P* = 0.0294, Figure 1E), although no difference was seen in the allele analysis (*P* = 0.0898, Figure 1F).

### Haplotypes and combinations of genotypes

**Figure 2:**
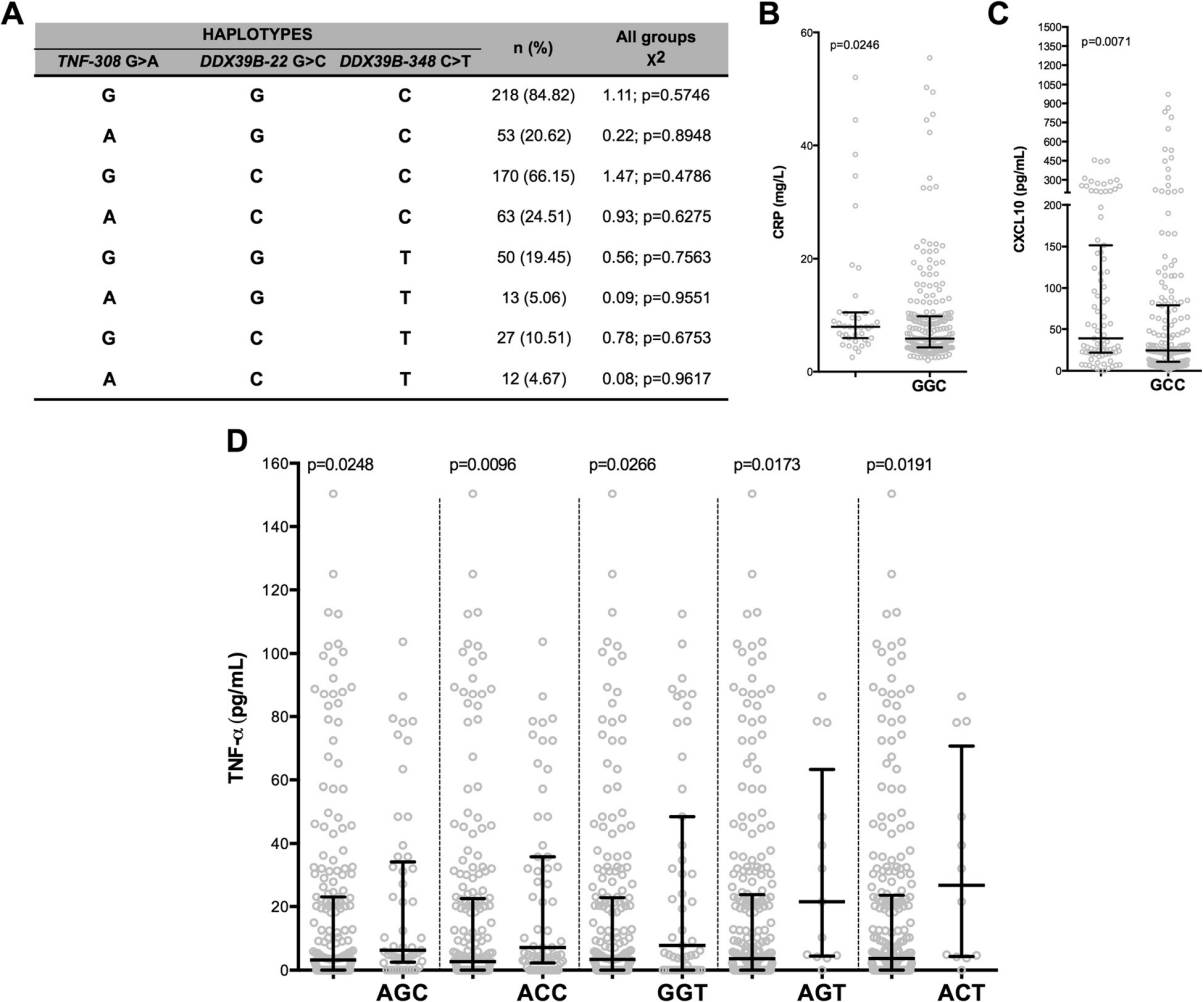
**Haplotype influence on inflammatory mediators and outcomes of malaria infection.** Each symbol represents a single patient, and the lines represent medians and interquartile range. **(A)** The figure represents all the possible haplotypes for *TNF*-308G > A, *DDX39B*-348C > T, and *DDX39B*-22C > G polymorphisms. Haplotypes were compared between the 3 categories of vivax malaria infections (uninfected, asymptomatic, mild malaria). **(B)** CRP levels in participants with GGC haplotype compared to individuals with other haplotypes. **(C)** CXCL10 plasma concentration in participants with GCC haplotype compared to individuals with other haplotypes. **(D)** TNF levels in participants with different haplotypes. Each haplotype was compared with participants with other haplotypes. The differences between groups illustrated in **(A)** were compared using the chi-square test. The data in **(B)**, **(C)**, and **(D)** were compared using the Mann–Whitney test. *P* values are shown in each panel.

**Figure 3:**
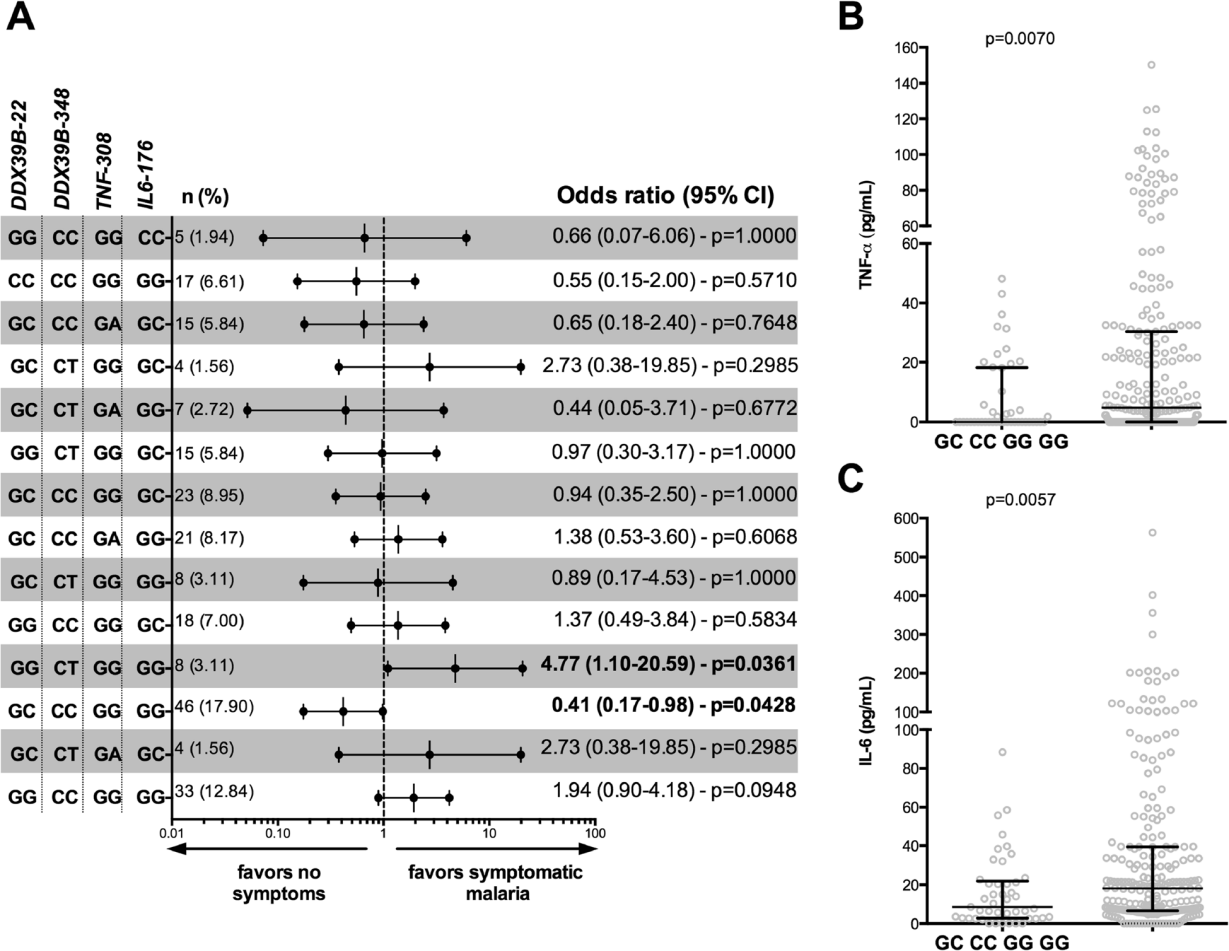
**Genotype combinations and outcomes of malaria infection. (A)** Univariate linear regression analysis of the different combination of *TNF*-308G > A, *IL6*-176G > C, *DDX39B*-348C > T, and *DDX39B*-22C > G genotypes. The odds ratios and respective 95% confidence intervals (95% CI) are shown according to each combination of genotypes compared with the other combinations. **(B-C)** Each symbol represents a single patient, and the lines represent medians and interquartile range. TNF and IL-6 levels in participants with the GC/CC/GG/GG combination compared with the other combinations. The data were compared using the Mann–Whitney test. *P* values are shown in each panel.

*DDX39B* and *TNF* are located in the same major histocompatibility complex (MHC) region on chromosome 6, and therefore assessment of the association of these polymorphisms with the outcomes of malaria can be performed using haplotype analysis. The assessment of haplotypes representing all possible combinations of *TNF*-308, *DDX39B*-22 and *DDX39B*-348 SNPs was unable to identify significant association with any of the clinical categories of vivax malaria infection (Figure 2A). Nevertheless, haplotypes were associated with differential expression of inflammatory mediators in plasma, which could potentially influence the degree of immunopathology and malaria manifestations. *TNF*-308, *DDX39B*-22, and *DDX39B*-348 haplotypes GGC and GCC were linked with lower concentrations of CRP and CXCL10, respectively, than other haplotypes (*P* = 0.0246, Figure 2B; *P* = 0.0071, Figure 3C; respectively). Intriguingly, several haplotypes were associated with increased plasma TNF levels (Figure 2D). Thus, because they were found to be associated with elevated or decreased levels of CRP, CXCL10, and TNF, haplotypes may be associated with the outcomes of vivax malaria infection.

Analysis of combinations of genotypes was performed to determine association with manifestations of vivax malaria. Participants with *DDX39B*-22/*DDX39B*-348/*TNF*-308/*IL6*-176 genotype combinations GC/CC/GG/GG and GG/CT/GG/GG had decreased and increased risk, respectively, of developing manifestations of malaria relative to asymptomatic participants (OR 0.41, 95% CI 0.17-0.98, *P* = 0.0428; OR 4.77, 95% CI 1.10-20.59, p = 0.0361; respectively, Figure 3A). Moreover, the genotype combination GC/CC/GG/GG was associated with lower TNF and IL-6 levels than other genotypes (*P* = 0.0070 and *P* = 0.0057, respectively; Figure 3B,C), suggesting that this set of genotypes may protect against more severe malaria because of association with reduced levels of inflammatory cytokines.

### Second series of study participants: *DDX39B* polymorphisms and complicated vivax malaria

An additional aim of this combined study was to specifically evaluate the association between *DDX39B* polymorphisms and complicated *P. vivax* malaria. Patients screened in a reference hospital from the Brazilian Amazon who manifested complicated malaria were compared with those presenting with mild disease. Participants with mild malaria had a median age of 36 years (IQR 27–46) and 81.16% were male (n = 56). Participants with complicated disease had a similar age (median 31 years, IQR 18–41; *P* = 0.0563) and there was a slightly lower proportion of males (19 of 31 [61.29%], *P* = 0.0460). Characteristics of the patients with complicated malaria are shown in Table 1. Interestingly, the patients in this study who were categorized with complicated vivax malaria exhibited more often hyperbilirubinaemia (serum total bilirubin >51.3 μmol/L), which has been reported to be a common complication of patients with vivax malaria [28,29].

There was significant difference in the distribution frequency of the polymorphism *DDX39B-*22C > G in the participants with mild versus complicated malaria (x2 = 6.72, *P* = 0.0347). The proportion of -22C homozygosity among patients with complicated and mild malaria was 25.80% (n = 8) and 7.25% (n = 5), respectively (Table 5). Participants were categorized based on the presence or absence of the G allele (genotypes GG and GC *vs* genotype CC) and the G allele was significantly more frequent in patients with mild vivax malaria than in patients with complicated infection (*P* = 0.0207, Table 5). No differences were seen for the polymorphism *DDX39B* (-348C > T) regarding the distribution of genotypes and alleles in patients with mild and complicated malaria (Table 5). The distribution of *DDX39B-*22C > G genotypes in the patients with mild malaria was not under HWE (x2 = 5.22, *P* = 0.0222). The *DDX39B* genotypes were under HWE in the patients with complicated malaria.

**Table 5:** ***DDX39B *** **polymorphisms (-22C > G and -348C > T) and complicated vivax malaria infections: second series of participants from Amazonas, Brazil**

	**Genotype**			**χ2**	**Allele frequency**		**χ2**	**CC *vs *** **CG + GG χ2**
	**CC n (%)**	**CG n (%)**	**GG n (%)**		**C (%)**	**G (%)**		
***DDX39B-22 *** **polymorphism**								
Mild malaria (n = 69)	5 (7.25)	41 (59.42)	23 (33.33)	**6.72 p = 0.0347**	51 (36.96)	87 (63.04)	p = 0.0628*	p = 0.0207*
Complicated malaria (n = 31)	8 (25.80)	16 (51.61)	7 (22.58)		32 (51.61)	30 (48.39)		
	**Genotype**				**Allele frequency**			**CC *vs *** **TC + TT χ2**
	**CC n (%)**	**TC n (%)**	**TT n (%)**		**C (%)**	**T (%)**		
***DDX39B-348 *** **polymorphism**								
Mild malaria (n = 69)	55 (79.71)	14 (20.29)	0 (0.00)	p = 7,850*^#^	124 (89.86)	14 (10.14)	p = 0.7965*	p = 7,850*
Complicated malaria (n = 31)	26 (83.87)	5 (16.13)	0 (0.00)		57 (91.93)	5 (8.07)		

*In these cases, Fisher exact test was used. ^#^Analysis excluded genotype TT. χ2: coefficient and *P* value measured using Chi-square test.

## Discussion

Immunity of the human host to malaria is likely to be mediated by T-cell recognition of *Plasmodium sp.* epitopes on infected host cells via class I and II MHC antigens [30]. Given the importance of the MHC to the immune response, genetic studies of the human MHC I have correlated polymorphisms in this region with susceptibility to malaria. The alleles A*30:01 and A*33:01 of MHC I were found to be associated with malaria severity in Mali [30-32]. The *DDX39B (BAT1)* gene is located on chromosome 6 near the *TNF* gene in the same MHC region and seems to influence expression of several immune-related genes [6]. This study found that the C allele of *DDX39B*-22C > G is a potential risk factor of complicated vivax malaria in the Brazilian Amazon. This finding may be expected, as this allele has been associated with reduced binding to transcription factors and expression of inflammatory cytokines [5]. Haplotype analysis (including *DDX39B* and *TNF* polymorphisms) found that genetic alterations in these immune-mediated genes may influence disease outcome by altering TNF plasma levels. In addition, the genotype combinations GC/CC/GG/GG and GG/CT/GG/GG, corresponding to the respective polymorphisms *DDX39B*-22/*DDX39B*-348/*TNF*-308/*IL6*-176, were associated with a decreased or increased risk, respectively, of developing mild vivax malaria, probably by altering TNF and IL-6 levels. To the best of our knowledge, this is the first report assessing the relationship between *DDX39B* polymorphisms and malaria outcomes, and also one of the few studies to analyze the manifestations of *P. vivax* infections in relation to combinations of immune-related genotypes.

Plasma concentrations of IL-6 have been found to be associated with severe disease and death from malaria, and the *IL6*-176C allele was associated with increased expression of IL-6 in neonates and adults developing acute phase reactions [14,15,33,34]. A study of sympatric ethnic groups in Mali found that the frequency of *IL6* CG/GG genotypes was higher in non-Fulani ethnic groups, who have increased susceptibility to malaria, in both symptomatic and asymptomatic falciparum malaria cases [35]. However, in our study, there were no differences in the distribution of *IL6*-176G > C in the participants making up the different clinical groups of vivax malaria, which may indicate that this polymorphism plays different roles in *P. vivax* infection and/or in a Brazilian population. Genetic changes in *TNF* have been described in several studies of different populations throughout the world, and there have sometimes been contradictory results [23]. The SNP *TNF*-308G > A has been associated with increased susceptibility, resistance or there has been no association with severity to malaria caused by *P. falciparum*[36-39]. A study of a population in the Brazilian Amazon that was similar to our population found that one *TNF* haplotype (*TNF*-1031T/-863A/-857T/-308G/-238G) including the *TNF*-308G allele was associated with increased susceptibility to mild vivax malaria [40]. Our study did not find an association between *TNF*-308G and clinical manifestations of malaria. It is noteworthy that *TNF*-308G > A may not be a causal mutation, and that this polymorphism could be in linkage disequilibrium with other causal mutations located close to the *TNF* gene [41]. Thus, this polymorphism may have a moderate effect or may have an epistatic effect on *DDX39B* mutations [42]. Polymorphisms in *DDX39B (BAT1)* have been described in several diseases with inflammatory profiles, including neuropathy, myasthenia gravis, allergies, Alzheimer disease, myocardial infarction, hepatitis, rheumatoid arthritis, Chagas disease, among others [25,43-48]. In this study it was found that the G allele of *DDX39B*-22C > G may be a resistance factor to malaria and the C allele a risk factor for disease complications. It has been reported that *DDX39B* promoter polymorphisms alter the binding of transcription factors (YY1 and Oct1) and may affect the transcription of this gene, and the sequences with -22G and -348 T alleles were expressed more efficiently than sequences containing -22C and-348C alleles [6]. An in vitro study found that BAT1 appeared to decrease the expression of TNF and IL-6 [5]; thus the G allele of *DDX39B*-22C > G, which enhances the expression of BAT1, may be protective against complicated malaria by decreasing the expression of proinflammatory cytokines.

Although the polymorphisms described in this study are not directly associated with the clinical manifestations of malaria, they can indirectly influence disease by altering the levels of inflammatory mediators involved in disease immunopathology. High plasma levels of TNF are related to the pathogenesis of signs associated with malaria, such as fever, and severe forms of infection, such as cerebral malaria and severe anemia [49]. Our study found that participants heterozygous (AG) for *TNF*-308G > A had higher plasma concentrations of TNF than homozygous participants with the wild-type (GG) polymorphism. The A allele of this SNP has been associated with increased production of TNF in several studies, and was often associated with the HLA-A1-B8-DR3 haplotype of the MHC region [50-53]. In this study results did not clearly demonstrate increased levels of TNF in participants homozygous for the A mutation, probably because of the small number of participants with this genotype who were recruited for the study. Furthermore, participants heterozygous (CT) for *DDX39B*-348C > T had higher plasma TNF than homozygous participants with the wild-type (CC) polymorphism, suggesting that an additional genetic mechanism appears to be associated with increased levels of this cytokine, and consequently the clinical outcome of malaria infection. CRP is an acute-phase inflammatory protein, and this study findings indicate that participants homozygous (CC) for *DDX39B*-22 (G > C) had increased levels of CRP, supporting an association of the C allele with risk of complicated malaria.

A single-point mutation is often not sufficient for predicting the susceptibility or resistance of individuals to malaria [4]. Another approach to investigating the differences in response to malaria infection is haplotype analysis of mutant alleles. *DDX39B* is situated in the central region of MHC on the short arm of human chromosome 6 and is approximately 150 kb from the *TNF* gene. The *NFKBIL1* gene, which encodes the inhibitor of κB-like protein (IκBL), a protein of unknown function, is situated between *DDX39B* and *TNF*[54]. Genetic variations in *NFKBIL1* are associated with susceptibility to inflammatory conditions such as periodontitis, chronic thromboembolic pulmonary hypertension, rheumatoid arthritis, and malaria [54-58]. Although *DDX39B* and *TNF* genes are near each other on the same chromosome and appear to influence the transcription of its gene products [5], this study did not identify a haplotype with *DDX39B* (22C > G and 348C > T) and *TNF*-308G > A polymorphisms that increased the risk of clincal vivax malaria. However, many haplotypes appear to markedly increase TNF levels, indirectly contributing to malaria susceptibility. It is noteworthy that conclusive findings from *DDX39B* and *TNF* haplotype analysis are limited by the considerable distance between these genes (approximately 150 kb). Similarly, it is reported here an analysis of susceptibility to clinical manifestations of malaria as a result of genotype combinations found that CG/CC/GG/GG and GG/CT/GG/GG, corresponding to the respective polymorphisms *DDX39B*-22/*DDX39B*-348/*TNF*-308/*IL6*-176, were associated with decreased and increased risk, respectively, of developing clinical manifestations of *P. vivax* infection. Intriguingly, *TNF*-308 and *IL6*-176 genotypes were wild-type homozygotes (GG for both) among the combinations, and changes were related to *DDX39B* genotypes. The substitution of *DDX39B* genotypes (-22 and -348) in the combinations completely changed the risk of developing manifestations of malaria, from susceptibility to resistance to illness from vivax malaria infections. These results lend support to the role of *DDX39B* as a regulatory gene that can alter transcription factors and inflammatory cytokines and influence the clinical outcome of inflammatory diseases [5,25]. Moreover, study participants with the genotype combination described here that was associated with resistance against manifestations of *P. vivax* infection (CG/CC/GG/GG) also had lower levels of proinflammatory TNF and IL-6, suggesting that *DDX39B* confers protection against malaria pathogenesis by reducing the inflammatory response.

This study was limited because of a small numbers of participants, which may have reduced its ability to detect significant differences between study groups. Indeed, the detection of a small difference (effect size) between groups at a significance level of 0.05 ideally would require at least 1,194 study participants. Therefore, our study results require validation in larger studies.

## Conclusion

Genetic alterations in the immune response against vivax malaria may predispose individuals to disease complications or protect them from clinical disease. The findings of this study provide support that the C allele of *DDX39B*-22C > G is a risk factor of complicated vivax malaria, and different haplotypes (including *DDX39B* and *TNF* polymorphisms) may influence disease outcomes by altering plasma levels of TNF. Moreover, the results suggest that combinations of genotypes (including *IL6*-176G > C) are associated with a decreased or increased risk of developing clinical manifestations of malaria and may also influence plasma TNF and IL-6 levels. Further prospective studies should be able to determine if these genetic determinants are critical for protection against the development of clinical *P. vivax* infection, and also identify individuals who are at risk of developing more complicated forms of malaria.

## Abbreviations

*DDX39B*: DEAD [Asp-Glu-Ala-Asp] box polypeptide 39B; BAT1: HLA-B associated transcript 1; RNA: Ribonucleic acid; TNF: Tumor necrosis factor; IL-6: Interleukin 6; SNP: Single nucleotide polymorphism; CXCL10: C-X-C motif chemokine 10; CRP: C-reactive protein; FUNASA: Brazilian National Foundation of Health; PCR: Polymerase chain reaction; FIOCRUZ: Oswaldo Cruz Foundation; HAV: Hepatitis A virus; HBV: Hepatitis B virus; HCV: Hepatitis C virus; HDV: Hepatitis D virus; HIV: Human immunodeficiency virus; FMT-HVD: Fundação de Medicina Tropical Dr Heitor Vieira Dourado; DNA: Deoxyribonucleic acid; OR: Odds ratio; CI: Confidence interval; HWE: Hardy-Weinberg equilibrium; IQR: Interquartile range; MHC: Major histocompatibility complex; IκBL: Inhibitor of κB-like protein.

## Competing interests

The authors declare that they have no competing interests.

## Authors’ contributions

VRRM performed the experiments, analyzed the data, and wrote the manuscript together with BBA and MBN. LCLS and GCG helped perform the experiments. BBA performed the field study and sampling in Rondonia and performed the plasma measurements and wrote the manuscript. BMLM was responsible for the field study and sampling in Manaus. MVGL supervised the clinical study and sampling in Manaus. MBN conceptualized the study, supervised the clinical study in Rondonia, and helped with data interpretation and the writing of the manuscript. All authors have read and approved the final version of the manuscript.
